# Distribution, Source, and Ecological Risk of Heavy Metals in Sewage Irrigation of Taiyuan, Shanxi Province, China

**DOI:** 10.3390/toxics12020120

**Published:** 2024-02-01

**Authors:** Ying Zhao, Han Yan, Fei Wang

**Affiliations:** 1 Department of Chemistry and Chemical Engineering, Jinzhong University, Jinzhong 030619, China; zhaoying@jzxy.edu.cn; 2 Shanxi Provincial Security Center of Ecological Environmental Monitoring and Emergency (Shanxi Provincial Academy of Ecological Environmental Science), Taiyuan 030027, China; sxstc13@126.com; 3 School of Physical Education, Shanxi University, Taiyuan 030006, China; 4 School of Life Science, Shanxi University, Taiyuan 030006, China

**Keywords:** heavy metal, agriculture, soil, sewage irrigation, ecological risk, Taiyuan city

## Abstract

The pollution of agricultural soil by heavy metals is a significant environmental issue that has a serious impact on human health and food security. This study focused on investigating the presence of heavy metal pollution in sewage-irrigated soils in Taiyuan city. A total of 110 soil samples were analyzed for the presence of As, Hg, Cd, Pb, Cr, Cu and Zn. The results showed that the concentrations of these metals ranged from 0.06 to 26.74 mg/kg for As, 0.00 to 0.84 mg/kg for Hg, 0.03 to 0.69 mg/kg for Cd, 44.32 to 100.09 mg/kg for Pb, 9.85 to 42.19 mg/kg for Cr, 13.38 to 53.72 mg/kg for Cu, and 42.77 to 145.47 mg/kg for Zn. The average concentrations of these metals were found to be below the risk values specified in the “Soil environmental quality (GB15618-2018)”, except for As and Cd in three sampling points in Xiaodian District. The heavy metal pollution in these areas can be attributed to various sources, such as industrial activities, the use of fertilizers and pesticides, and the irrigation process. According to the geo–accumulation index, the agricultural soil in the Taiyuan irrigation area was found to be uncontaminated by Zn, Cr, Cu, and As, and lightly contaminated by Cd, Hg, and Pb. The Nemerow Pollution Index indicated that the soil at all sampling points exhibited a slight level of pollution. Moreover, the ecological risk assessment indicated that all heavy metals posed a slight level of pollution. The findings of this study provide a scientific basis for the development of effective policies and measures for soil environmental protection and pollution control.

## 1. Introduction

Because of global water scarcity, the amount of freshwater water allocated to farmland is decreasing. As a result, sewage has become a source of irrigation water [1,2]. Sewage irrigation has both benefits and drawbacks. On one hand, it brings water, nutrients, and organic matter to the soil. On the other hand, it also contains harmful substances like heavy metals [3]. If sewage irrigation is practiced over a long period of time, it can lead to the accumulation of heavy metals in agricultural soil [4,5]. This accumulation is typically found in the surface layer of 0–20 cm [6]. Moreover, crops and vegetables have the ability to actively or passively absorb these heavy metals, which can eventually concentrate in the edible parts [7,8].

Generally, heavy metal contents vary between regions and depend on the source and volume of wastewater and its composition. Jabeen et al. [9] concluded that irrigation with sewage water is the main factor contributing to the accumulation of heavy metals, making vegetables unfit for human consumption. Sayo et al. [10] revealed that continuous use of sewage effluent for irrigation could gradually lead to the accumulation of heavy metals in the soil, which could eventually result in increased uptake of these heavy metals by the different parts of growing vegetables. Othman et al. [11] assessed the effect of wastewater reuse in irrigation on soil and crops. Based on their meta analysis, the results showed that the concentration of heavy metals in the edible parts of vegetables increased by 3–9 times compared to those irrigated with fresh water. Wang et al. [12] determined the contents of heavy metals in agricultural soil collected from a sewage irrigation area in Wuqing District, Tianjin. They found higher levels of Cd and Pb, indicating slight soil pollution. Singh [13] studied wastewater irrigation worldwide and indicated that untreated wastewater caused the deposition of heavy metals in soils and plants, posing a threat to human health. On the other hand, the identification and risk assessment of heavy metals are crucial for controlling regional pollution. The Geoaccumulation Index [14,15], Nemerow Comprehensive Index [16,17], and Potential Ecological Risk Index [18,19] methods have been widely used for evaluating pollution in soil ecological risk assessments. Although wastewater is an essential source for irrigating agricultural crops, potential environmental and human health risks are associated with the reuse of this water due to the accumulation of heavy metals. In fact, the accumulation of wastewater contaminants, including heavy metals, in the environment is inevitable. The contamination of toxic metals negatively impacts all environmental constituents, especially agricultural soil ecosystems [20]. It is clear that environmental issues influenced by heavy metal pollution should be emphasized in order to make wastewater reuse sustainable and reduce adverse environmental impacts. However, there is limited information on soil heavy metal contamination in sewage irrigation areas in the northern regions, where heavy industries are the primary sources.

Shanxi Province is the national energy and heavy chemical industry base, and it is also the largest coke production base in China [21,22]. As a result, heavy metal pollution of the soil in this region severely affects the surrounding ecological environment, especially causing serious pollution to the agricultural ecosystem. Taiyuan City, the capital city of Shanxi Province, is an industrialized city with a primary focus on the coal and machinery industries. Since the 1970s, Taiyuan City has been using sewage irrigation due to a severe shortage of water resources. In addition to being readily available, sewage irrigation is also cost–effective. However, the main pollutants from sewage irrigation accumulate in the irrigation areas, posing a significant risk to the ecosystem and human health, given the rich resources and high fertility of the sewage used for irrigation. Therefore, it is important to study the soil environmental quality in sewage irrigation areas to ensure the safety of the living environment in Shanxi Province and the health of its residents.

The objectives of this study were to (1) investigate the levels of contamination and the spatial distribution of arsenic (As), mercury (Hg), cadmium (Cd), lead (Pb), chromium (Cr), copper (Cu), and zinc (Zn) in agricultural soil within the sewage irrigation area; (2) to determine the primary sources of contamination using multivariate statistical methods; and (3) to assess the ecological risks using the Geoaccumulation Index (GAI), Nemerow Pollution Index (NPI), and Potential Ecological Risk Index (PERI).

## 2. Materials and Methods

### 2.1. Study Area and Sample Collection

The sewage irrigation area in Taiyuan City covers approximately 270 km^2^, mostly in the northern (Jiancaoping Area) and southern (Xiaodian Area, Jinyuan Area) parts of Taiyuan City, as well as in Qingxu County. The largest sewage irrigation area is found in the southern districts and counties [23]. For this study, the focus areas were Xiaodian District, Jinyuan District, and Qingxu County. In Xiaodian District, there are three main irrigation channels: the Donggan Canal of the Fenhe River Dam, the Beizhang waste canal, and the Taiyu waste canal. These channels receive both agricultural irrigation sewage and untreated industrial wastewater. The main crops grown in Xiaodian District are corn, wheat, and vegetables. In Jinyuan District, the primary irrigation channel is the Xigan Canal. The water in this canal mainly comes from the Fenhe River and wastewater from local industrial and mining enterprises, with a smaller amount of domestic sewage. The wastewater contains pollutants such as mercury, chromium, sulfides, and other substances. Corn and sorghum grown in Jinyuan District are exclusively irrigated with this wastewater. In Qingxu County, the main irrigation channels are the Donggan Canal and Xigan Canal. The sources of pollution in this area are mainly small amounts of domestic and industrial wastewater. Irrigation takes place once each spring and winter, and the main economic crops grown are corn and sorghum. 

**Figure 1:**
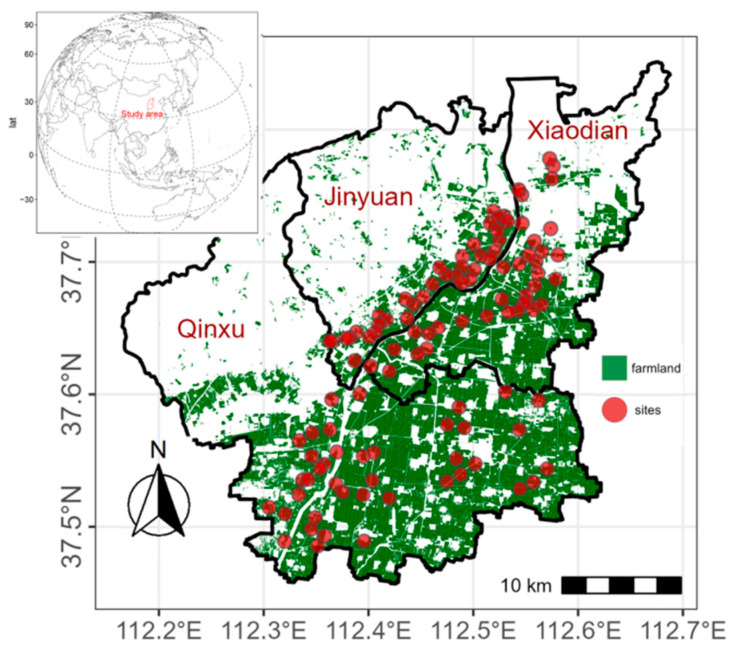
Distribution of the sampling sites in the sewage irrigation area of Taiyuan.

The grid-point method was utilized for sample placement alongside the implementation of the GPS positioning system. Various factors were considered, such as the distribution and flow direction of irrigation channels, sewage water quality, soil types, and the history of sewage irrigation. A total of 110 sampling points were established, comprising of 35 points in Xiaodian Area, 35 points in Jinyuan Area, and 40 points in Qingxu County (Figure 1). Topsoil samples (0–20 cm) were collected in June 2020 using a plastic spade, carefully stored in PVC packages with labels, and subsequently transported to the laboratory. Following this, the soil samples were dried indoors, grinded with a porcelain mortar, sieved through a size 100 mesh, and stored in plastic bags.

### 2.2. Sample Analysis

Seven heavy metals (As, Hg, Cd, Pb, Cr, Cu and Zn) were detected in the soil. For the measurement of Cd, Pb, Cr, Cu and Zn contents, a 0.1 g sample was heated and digested with an acid mixture (HNO_3_ + HCl + HF + H_2_O_2_) until only a negligible amount of white residue remained. The solution was then evaporated, extracted, and sieved through a Whatman paper. The concentrations of Cd, Pb, Cr, Cu and Zn were analyzed using an Agilent 7700e ICP–MS instrument [24]. The detection limits for Cd, Pb, Cr, Cu and Zn were 0.6, 2.1, 1.0, 1.2 and 3.2 mg/kg, respectively. For the analysis of Hg and As concentrations, a 0.5 g sample was weighed and heatedly digested with a mixture of HNO_3_ + HCL. The contents were then measured using a Haiguang AFS–9760 atomic fluorescence spectrophotometer from China [25]. The detection limits were 0.01 mg/kg for As and 0.002 mg/kg for Hg, respectively.

The Quality Control/Assurance (QC/QA) procedure was implemented to ensure the data’s representability, accuracy, and comparability. An analytical blank and spiked sample were analyzed after every 30 samples. The blank determination results were all below the method detection limit. The calibration curve’s correlation coefficient exceeded 0.999. The standard recovery rates for Cd, Pb, Cr, Cu, and Zn ranged from 75% to 125% [26]. 

### 2.3. Ecological Risk Assessment

The Geoaccumulation Index (*I_geo_*), also known as the Muller Index, is a widely used measure for assessing heavy metal pollution in sediment and other environments. It considers both the influence of background values resulting from natural geological processes and the impact of human activities on heavy metal pollution. By incorporating both natural distribution and human activities, the index serves as a crucial parameter for distinguishing the effects of human activities [27,28]. The expression for the index is as follows:

(1) $$I_{geo}=\log_{2}\left[\frac{C_{i}}{1.5B_{i}}\right]$$

where *C_i_* (mg/kg) represents the measured concentration of a specific heavy metal, while *B_i_* (mg/kg) represents the geochemical background value of the element. In this study, Taiyuan city had the following corresponding values: 7.60, 0.03, 0.08, 57.30, 13.80, 18.40, and 56.30 mg/kg for As, Hg, Cd, Cr, Pb, Cu and Zn, respectively. The constant 1.5 accounts for the natural fluctuations of the metal in soil. The *I_geo_* is classified into seven grades (0 to 6): *I_geo_* < 0 indicates an unpolluted level, 0 ≤ *I_geo_* < 1 represents lightly polluted level, 1 ≤ *I_geo_* < 2 indicates a moderately polluted level, 2 ≤ *I_geo_* < 3 represents a moderately to heavily polluted level, 3 ≤ *I_geo_* < 4 indicates a heavily polluted level, 4 ≤ *I_geo_* < 5 represents a heavily to extremely polluted level, and *I_geo_* ≥ 5 represents an extremely polluted level.

The Nemerow Pollution Index (NPI) is a comprehensive index used to evaluate the pollution caused by heavy metals [29,30]. It considers not only the overall pollution caused by these metals, but also gives importance to the most severe pollution. In doing so, it avoids any bias that may be introduced by subjective factors in the weighting process. Consequently, this method overcomes the limitations of the average method when distributing various heavy metals in this study. The calculation formula is provided as follows:

(2) $$P_{N}=\sqrt{\frac{{\left(\frac{C_{i}}{S_{i}}\right)}_{mean}+{\left(\frac{C_{i}}{S_{i}}\right)}_{\max}}{2}}$$

where *P_N_* represents the NPI, *C_i_* (mg/kg) represents the monitored content, and *S_i_* (mg/kg) represents the reference value, which is quoted from the criterion “Soil environmental quality: Risk control standard for soil contamination of agricultural land (GB15618-2018)”. The standard values for As, Hg, Cd, Cr, Pb, Cu and Zn are 25, 3.4, 0.6, 250, 170, 100 and 300 mg/kg, respectively [31]. The *P_N_* can be defined as follows: *P_N_* ≤ 0.7 represents clean; 0.7 < *P_N_* ≤ 1 is a warning limit; 1 < *P_N_* ≤ 2 is slightly polluted; 2 < *P_N_* ≤ 3 is moderate polluted; and *P_N_* > 3 is heavily polluted [32,33].

The Potential Ecological Risk Index (PERI) is calculated to evaluate the ecological and toxicological effects of heavy metals in soil. This index quantitatively classifies the potential hazards of heavy metals [34]. It takes into account the content of heavy metals in the soil to reflect the impact of each pollutant in a specific environment. Additionally, it combines the ecological effects, environmental effects, and toxicology of heavy metals to reveal the comprehensive impact of various pollutants. The PERI equation is used to classify the degree of potential ecological risks of heavy metals. The PERI equation is provided as follows:

(3) $$PERI=\sum_{i}^{n}E_{r}^{i}=\sum_{i}^{n}\left(T_{i}\times \frac{C_{i}}{S_{i}}\right)$$

where *C_i_* and *S_i_* represent the monitored and standard values, respectively. The single ecological risk index, *E_r_*, is classified as follows: $E_{r}^{i}$ < 40 indicates low pollution; 40 ≤ $E_{r}^{i}$ < 80 indicates moderate pollution; 80 ≤ $E_{r}^{i}$ < 160 indicates considerable pollution; 160 ≤ $E_{r}^{i}$ < 320 indicates strong pollution; and 320 ≤ $E_{r}^{i}$ indicates extreme pollution. The toxic response factor, *T_i_*, for each metal is as follows: Cd = 30, Hg = 40, As = 10, Pb = 5, Cr = 2, Cu = 5, and Zn = 1. The *PERI* classification is as follows: *PERI* ≤ 150 indicates a low level, whereas 150 < *PERI* ≤ 300, 300 < *PERI* ≤ 600, and *PERI* > 600 indicate a moderate, considerable, and strong level, respectively [35,36].

### 2.4. Statistical Method

All statistical analyses and diagrams were conducted using R (version R 4.2.0, https://cran.r-project.org, assessed on 20 November 2023). The data cleaning (i.e., removing missing values) and collation (i.e., deriving variables) were performed using the “tidyverse” package. Descriptive statistics (including the maximum, minimum, mean, and variation coefficient in Table 1) and correlation calculation (indicating the pairwise correlation of seven heavy metal contents) were found using the “gtsummary” package. The principal component analysis was performed using the PCA method with the “rstate” package in R. The spatial interpolation of IDW was carried out using the ”sf ”package and “rspatial” package. All plots were drawn based on the “ggplot2” package of R.

## 3. Results and Discussion

### 3.1. Distribution of Heavy Metals in the Study Area

**Figure 2:**
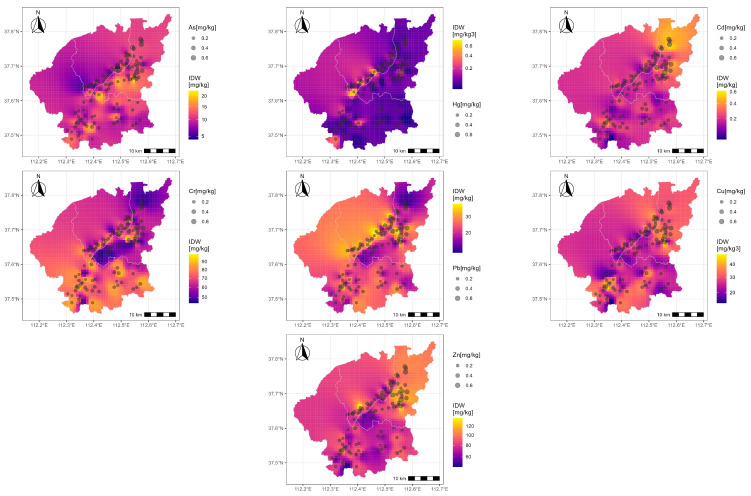
Concentrations and distribution of seven heavy metals in different area.

The distribution of heavy metals varies significantly across different regions. According to monitoring and analysis results, the contents of As, Hg, Cd, Cr, Pb, Cu, and Zn ranged from 0.06 to 26.74, 0.00 to 0.84, 0.03 to 0.69, 44.32 to 100.09, 9.85 to 42.19, 13.38 to 53.72, and 42.77 to 145.47 mg/kg, respectively (Table 1). The spatial distribution of these metals can be summarized as follows: Xiaodian District > Jinyuan District > Qingxu County. Specifically, the Cd content in Xiaodian District was significantly higher than in Jinyuan District and Qingxu County, while the Hg and Pb contents in Jinyuan District were significantly higher than in Xiaodian District and Qingxu County (*p* < 0.05). Figure 2 shows the concentration variation of heavy metals with distances using Inverse Distance Weight interpolation. This method was based on data from 114 sampling sites, which ensured the accuracy. Therefore, this figure reveals the overall variation trend in each area. Similar methods were also used by Li et al. [8] and Yang et al. [37]. As shown in Figure 2, the Xiaodian Area had higher As, Cd, and Cu contents in the south, north, and southeast parts, respectively. The Jinyuan Area had higher Hg and Cu contents, while the Qingxu Area had higher Cr content. Additionally, Hg had the largest coefficient of variation at 129.85%, while Cr had the smallest value at 20.58%. The average values of the heavy metals were ordered as follows: Hg > Cd > As > Pb > Cu > Zn > Cr. Overall, the variation coefficients of the heavy metals were relatively small, except Hg, indicating a generally low variation in heavy metal concentrations among sampling points.

When comparing this study with other studies (Table 2), the measured data were much lower than those observed in the farmland soils of Hunan Province and Pakistan [38,39]. However, the contents of six heavy metals were higher than those found in the agricultural soils in Guangdong, except for Pb [40]. The heavy metal levels in Taiyuan irrigation area were almost the same as those in Tianjin, Qatar, and Shandong [12,41,42]. Therefore, the heavy metal contents in Taiyuan irrigation area were relatively low.

### 3.2. Source Analysis of the Heavy Metals

The total amount of heavy metal elements in the soil showed a correlation due to similar geochemical conditions and the presence of metal elements from pollution sources, leading to soil contamination. The significant and highly significant correlations among these elements indicate a shared origin or complex pollution [43]. The analysis results from this study (Table 3) revealed significant correlations between Pb, Zn, Cu, and Cd, implying that these elements may have similar sources. However, Hg showed less correlation with the other elements.

The standardized data underwent PCA analysis, resulting in matrix eigenvalues, contribution rates, and cumulative contribution rates. Data standardization involves transforming data into relative indicators to allow for comparison and to eliminate the measurement inconsistency caused by different units. It can be achieved by dividing the difference by the standard deviation, where the difference is the monitoring value minus the average value. Following the criterion of eigenvalues ≥ 1, two principal components were extracted from the monitoring values of Xiaodian District (Table 4). The contribution rate of the first principal component (PC1) was 43.04%, indicating that 43.04% of the overall information was contained, mainly involving the indicators Cr, Cu, Zn, and Pb. The contribution rate of the second principal component (PC2) was 21.89%, representing a comprehensive reflection of As, Cd, and Hg. Therefore, Cr, Cu, Zn, and Pb were the major pollution factors in Xiaodian District. Pb, Hg, Cr and Cd are commonly found in catalysts and additives used in rubber production. Additionally, Cu, Cr, Zn and As in dye structures have significant biological toxicity and severely pollute the soil environment [44]. Thus, the possible sources may be related to the wastewater discharge from Taiyuan Rubber Factory and Shanxi Knitting Factory in the Xiaodian Area. In the southeast direction, the concentrations of Cd, Zn, and Pb in Xiaodian District were higher than in Jinyuan District, consistent with the prevailing local wind direction. This suggests that dry and wet deposition of Cd, Zn, and Pb pollutants in the atmosphere are also important factors contributing to the more severe pollution in Xiaodian District. Similar reasons were also found by Zhang et al. [45]. Three principal components were extracted from the heavy metal concentrations of Jinyuan District (Table 4). The contribution rate of the first PC was 25.459%, mainly involving the indicators Cd, As, and Zn, which were the main pollution factors. It is reported that common heavy metal pollutants in wastewater from industrial and thermal power plants include Pb, Hg, Cd, Cr and others [46]. Therefore, the discharges of related enterprises, including Taiyuan Chemical Factories and Taiyuan First Thermal Power Plant, were the sources of pollution in Jinyuan District. This may be related to sewage discharge from dyeing and printing factories. As for the monitoring data in Qingxu County, three principal components were extracted (Table 4). The contribution rate of the first PC was 42.70%, and Cd, Pb, Cr, Cu, and Zn were the primary indicators, which might be related to the discharges from industrial parks in this area. Moreover, Salem et al. assessed the effect of phosphate and urea fertilizers on heavy metal contents in agricultural soils [47]. The results showed that despite the application of phosphate and urea fertilizers for over forty years, the concentration of Cr, Cu, Cd, Mn, Zn, Ni, Fe and Pb remained below the permissible limits set by the World Health Organization/Food and Agriculture Organization. Thus, the use of phosphate and urea fertilizers does not have a detrimental effect on heavy metal pollution. However, as traditional pesticides and fertilizers often contain As, Cu, and Zn, the indiscriminate use of these pesticides could lead to their accumulation in the soil. The heavy metal concentrations in the Xiaodian, Jinyuan, and Qingxu areas were 1.01–3.40, 1.22–5.31, and 1.31–2.32 times higher, respectively, than the background values of soil in Taiyuan City. Based on this, there is virtually no background effect from the existing soil.

To summarize, the main source of heavy metal content in the farmland soil of the Taiyuan sewage irrigation area is primarily wastewater, with a smaller portion coming from pesticides and fertilizers that contain heavy metals.

### 3.3. Ecological Risk Assessment

**Figure 3:**
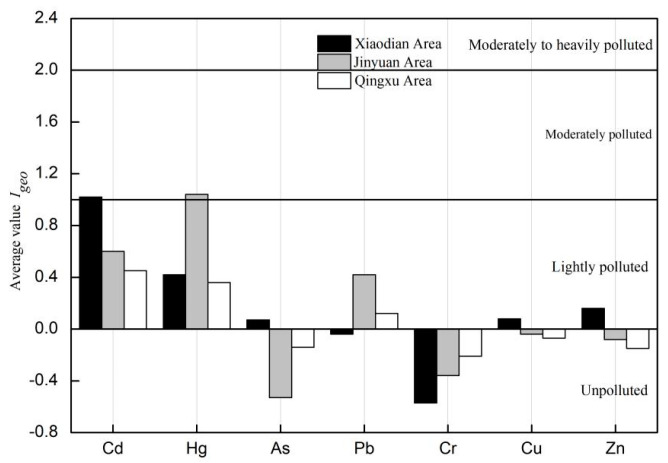
Geo−accumulation index of seven heavy metals in the study area.

Based on the analysis of the geo-accumulation index evaluation (Figure 3), the soil in Xiaodian District was found to be polluted with heavy metals in the following order: Cd > Hg > Zn > Cu > As > Pb > Cr. Pb and Cr are categorized as non-polluted, while Hg, Zn, Cu, and As show varying levels of pollution from non-polluted to moderate. Cd exhibits a moderate level of pollution. In Jinyuan District, the pollution levels are ordered as follows: Hg > Cd > Pb > Cu > Zn > Cr > As. In this district, Cr, Zn, Cu, and As show non-polluted levels, while Pb and Cd show non-polluted to moderately polluted levels, and Hg demonstrates a moderate level of pollution. As for Qingxu County, the heavy metal pollution levels were found to be in the following order: Cd > Hg > Pb > Cu > As > Zn > Cr. In this case, Cr, Zn, Cu, and As show non-polluted levels, while Cd, Hg, and Pb exhibit non-polluted to moderately polluted levels. Comparing the different regions, the average geo-accumulation index for Cd, Cu, and Zn indicates a higher level in Xiaodian District > Jinyuan District > Qingxu County. Hg and Pb are characterized by higher contents in Jinyuan District. In summary, the average geo-accumulation index values for Cd and Hg are relatively high, indicating non-polluted to moderately polluted or moderately polluted conditions. Xiaodian District has the highest values for four heavy metals, while Jinyuan District has the maximum contents for two heavy metals.

As demonstrated in Table 5, only the *I_geo_* value of Cd in Xiaodian District and the Hg accumulation index in Jinyuan District exceeded 1, indicating moderate pollution. However, certain individual locations exhibited pollution levels higher than moderate. In Xiaodian District, 25.71% of the sampled points showed moderate pollution levels for Cd, and 8.75% of the points reached a moderate to strong pollution level. For Hg, 25.71% of the sampled points indicated moderate pollution, while 2.86% of the points fell into the moderate to strong pollution category. In Jinyuan District, 25.71% of the sampled points experienced moderate Cd pollution, and 28.57% of the points displayed moderate Hg pollution. Moreover, 5.71% of the points in both districts exhibited moderate to strong pollution, strong pollution, and strong to extremely strong pollution. In Qingxu County, 20.00% of the sampled points showed moderate Cd pollution, and even 2.86% of the points reached a moderate to strong pollution level. As for Hg, 8.57% of the sampled points reached a moderate pollution level, and an additional 8.57% of the points reached a moderate to strong pollution level. The Pb accumulation index indicated moderate pollution at 2.86% of the points. In summary, for the Taiyuan irrigation area, the *I_geo_* values for Cd, Hg, As, Pb, Cr, Cu and Zn were 0.68, 0.60, −0.20, 0.16, −0.37, −0.01 and −0.03, respectively. Thus, Cd, Hg and Pb appeared to have slightly polluted the area.

The evaluation results of the NPI in the current study show that the average values for Xiaodian District, Jinyuan District, and Qingxu County are 0.42, 0.31, and 0.32, respectively. All of these values fall within the non-polluted range. Specifically, the soil heavy metal pollution in Jinyuan District and Qingxu County is considered safe, with NPI ranges of 0.21–0.44 and 0.21–0.63, respectively (Table 6). The comprehensive PERI evaluation results indicate that the average values for Xiaodian District, Jinyuan District, and Qingxu County are 22.32, 18.2, and 17.13, respectively. These values all fall within the low pollution level. The ranges of PERI for these three areas are 15.65–46.67, 11.82–23.32, and 8.77–36.45, all maximum values below 150. Therefore, the soil at all sampling points is classified as having a slight pollution level.

**Figure 4:**
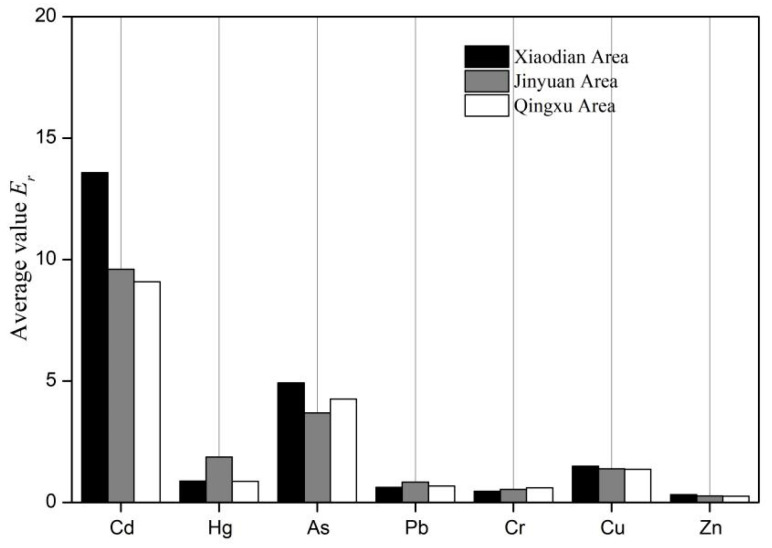
Average single ecological risk index in different areas.

The single-factor PERI indicated that the E_r_ values of s heavy metals were below 40 at all sampling points, indicating slight pollution (Table 7). Therefore, the potential risk for each individual heavy metal was relatively low. The comprehensive PERI showed that Xiaodian District, Jinyuan District, and Qingxu County had average values of 22.32, 18.2, and 17.13, respectively, all falling within the low pollution level (Figure 4). The ranges of PERI for these three areas were 15.65–46.67, 11.82–23.32, and 8.77–36.45, with all maximum values below 150. Consequently, the soil at all sampling points can be classified as slightly polluted. Overall, the potential ecological risks were relatively high in Xiaodian and Jinyuan Districts, while in Qingxu County, the risk was lower but still showed increasing pollution.

To summarize, Xiaodian District has higher pollution levels compared to Jinyuan District and Qingxu County, where pollution is relatively lighter. However, there is an overall trend of increasing pollution. This study reveals that the sewage in irrigated areas is mainly composed of domestic and some industrial wastewater, which contains significant amounts of heavy metals. These heavy metals enter the soil when the sewage is used for irrigation. Furthermore, improper use of pesticides and fertilizers, along with human activities, worsens heavy metal pollution in local farmland. It is crucial to pay attention to the increasing levels of heavy metal pollution in the Taiyuan sewage irrigation area. Additionally, pollution sources should be identified and measures should be taken to prohibit sewage irrigation and the use of heavy metal-containing fertilizers. It is also important to avoid the use of pesticides and implement changes in planting structure, such as cultivating economic crops like seedlings, flowers, and fruit trees in heavily polluted areas. In moderately to mildly polluted areas, planting low-accumulation crops or conducting soil heavy metal remediation under suitable conditions is recommended.

## 4. Conclusions

This research investigates the pollution characteristics of heavy metals in the soil of a sewage irrigation area. The study compares the contents, determines the sources, and assesses the ecological risk. The main conclusions are as follows:

(1) The average contents of seven heavy metals are as follows: Zn > Cr > Cu > Pb > As > Cd > Hg. The spatial distribution shows the following trend: Xiaodian District > Jinyuan District > Qingxu County. According to the “Soil environmental quality (GB15618-2018)”, the average content of the seven heavy metal elements does not exceed the risk values, except for the As and Cd contents in three sampling points of Xiaodian District.

(2) The PCA results displayed that the main pollutants were Cd, Cr, Cu, Zn and Pb, which primarily originated from the wastewater discharged by industrial and mining enterprises, as well as the use of chemical fertilizers and pesticides.

(3) The *I_geo_* index of heavy metals in farmland soil of the irrigation area of Taiyuan suggests the accumulation of Pb, Cd, and Hg, which belong to lightly polluted levels. The Nemerow pollution index results indicate that the average *P_N_* values for Jinyuan District and Qingxu County soils are both less than 1, classifying them as safe. However, in Xiaodian District, 8.57% of soil samples fall into the caution level. The single-factor PERI for individual heavy metals shows that the average *E_i_* values in all three areas are less than 40, implying a low potential risk. The average values of the PERI are 22.32, 18.2, and 17.13, respectively, indicating a slight pollution level. Overall, the potential ecological risk of the soil was observed in the following order: Jinyuan District > Xiaodian District > Qingxu County, with Cd and Hg showing higher pollution levels compared to other elements.

The discharges of urban industrial and enterprises presented significant potential ecological risks from heavy metals in the soil. Thus, these areas should receive additional attention.

## Figures and Tables

**Table 1:** Comparison of soil heavy metals content between this study and other areas.

District		As	Hg	Cd	Cr	Pb	Cu	Zn
Xiaodian Area	Max. (mg/kg)	26.74	0.34	0.69	100.09	42.19	50.84	144.13
	Min. (mg/kg)	7.80	0.02	0.11	44.32	9.85	18.36	55.00
	Mean (mg/kg)	12.33	0.07	0.27	59.15	21.52	30.03	97.18
	Variation coefficient (%)	27.90	77.16	51.22	22.65	39.25	23.21	24.87
Jinyuan Area	Max. (mg/kg)	14.63	0.84	0.30	92.12	39.82	41.68	145.47
	Min. (mg/kg)	0.06	0.001	0.10	48.11	15.60	15.00	56.35
	Mean (mg/kg)	9.22	0.16	0.19	67.91	28.42	27.87	81.77
	Variation coefficient (%)	33.97	127.3	31.71	18.96	23.63	27.90	21.34
Qingxu Area	Max. (mg/kg)	18.18	0.34	0.54	96.68	42.02	53.72	124.74
	Min. (mg/kg)	7.04	0.02	0.03	47.96	15.94	13.38	42.77
	Mean (mg/kg)	10.64	0.07	0.18	75.20	22.99	27.49	77.54
	Variation coefficient (%)	26.00	99.46	44.95	14.53	22.39	29.35	18.68

**Table 2:** Comparison of soil heavy metal contents between this study and other areas.

Site	Cd	Hg	As	Pb	Cr	Cu	Zn	Reference
	mg/kg							
Qidong County, Hunan	10.50	0.45	105.02	92.70	100.52	62.56	517.20	[38]
Vehari, Pakistan	1.76			34.00	65.60	56.00	89.9	[39]
Fogang County, Guangdong	0.07	0.10	5.30	51.87	27.49	12.15	56.34	[40]
Wuqing, Tianjin	0.26	-	-	29.92	78.99	25.14	79.57	[12]
Qatar	0.20	-	27.60	18.20	85.70	25.60	92.40	[41]
Longkou, Shandong	0.20	-	7.96	35.08	-	35.3	77.89	[42]
This research	0.21	0.10	10.73	24.25	67.77	28.42	85.13	-

**Table 3:** Pearson coefficients of different heavy metals.

	As	Hg	Cd	Cr	Pb	Cu	Zn
As	1	−0.030	−0.106	−0.202 *	−0.283 *	0.059	0.013
Hg		1	−0.086	0.232 *	0.233 **	−0.076	−0.037
Cd			1	0.058	0.085	0.369 **	0.498 **
Cr				1	0.296 **	0.270 **	0.091
Pb					1	0.300 **	0.251 *
Cu						1	0.392 **
Zn							1

Note: significant statistical level * *p* < 0.05; ** *p* < 0.01.

**Table 4:** Principal components of different areas.

Element	Xiaodian Area		Jinyuan Area			Qingxu Area		
	PC1	PC2	PC1	PC2	PC3	PC1	PC2	PC3
Cd	0.157	0.825	−0.777	0.152	0.114	0.865	−0.05	−0.127
Hg	0.256	0.39	0.528	0.553	−0.344	0.034	0.942	0.009
As	−0.075	−0.736	0.705	−0.134	0.516	−0.022	0.023	0.968
Pb	0.869	0.131	0.183	−0.327	−0.782	0.699	0.049	0.158
Cr	0.86	0.144	−0.03	0.915	0.06	0.779	0.114	−0.205
Cu	0.877	0.27	0.156	−0.291	0.577	0.767	−0.395	−0.016
Zn	0.808	0.215	−0.586	−0.183	−0.046	0.746	0.392	0.183

**Table 5:** Geo-accumulation index assessment of the sampling points in different areas.

Study Area	Element	Average *I_geo_*	Sampling Points (%)						
			*I_geo_* ≤ 0	0 < *I_geo_* ≤ 1	1 < *I_geo_* ≤ 2	2 < *I_geo_* ≤ 3	3 < *I_geo_* ≤ 4	4 < *I_geo_* ≤ 5	*I_geo_* > 5
Xiaodian area	Cd	1.02	2.86	62.86	25.71	8.57	0.00	0.00	0.00
	Hg	0.42	40.00	31.43	25.71	2.86	0.00	0.00	0.00
	As	0.07	37.14	60.00	2.86	0.00	0.00	0.00	0.00
	Pb	−0.04	62.86	34.29	2.86	0.00	0.00	0.00	0.00
	Cr	−0.57	97.14	2.86	0.00	0.00	0.00	0.00	0.00
	Cu	0.08	31.43	68.57	0.00	0.00	0.00	0.00	0.00
	Zn	0.16	25.71	74.29	0.00	0.00	0.00	0.00	0.00
Jinyuan area	Cd	0.60	11.43	62.86	25.71	0.00	0.00	0.00	0.00
	Hg	1.04	17.14	37.14	28.57	5.71	5.71	5.71	0.00
	As	−0.53	74.29	25.71	0.00	0.00	0.00	0.00	0.00
	Pb	0.42	8.57	91.43	0.00	0.00	0.00	0.00	0.00
	Cr	−0.36	88.57	11.43	0.00	0.00	0.00	0.00	0.00
	Cu	−0.04	48.57	51.43	0.00	0.00	0.00	0.00	0.00
	Zn	−0.08	57.14	42.86	0.00	0.00	0.00	0.00	0.00
Qingxu area	Cd	0.45	20.00	71.43	20.00	2.86	0.00	0.00	0.00
	Hg	0.36	40.00	57.14	8.57	8.57	0.00	0.00	0.00
	As	−0.14	74.29	40.00	0.00	0.00	0.00	0.00	0.00
	Pb	0.12	45.71	65.71	2.86	0.00	0.00	0.00	0.00
	Cr	−0.21	97.14	17.14	0.00	0.00	0.00	0.00	0.00
	Cu	−0.07	65.71	48.57	0.00	0.00	0.00	0.00	0.00
	Zn	−0.15	77.14	37.14	0.00	0.00	0.00	0.00	0.00

**Table 6:** Nemerow Pollution Index assessment of the study areas.

Location	*P_N_*	$\overset{—}{P_{N}}$	Pollution Level
Xiaodian District	0.24–0.81	0.42	Clean
Jinyuan District	0.21–0.44	0.31	Clean
Qingxu District	0.21–0.63	0.32	Clean

**Table 7:** The range of single-factor PERI in different areas.

*E_r_*	Xiaodian Area	Jinyuan Area	Qingxu Area
Cd	5.50–34.25	5.02–14.98	1.32–26.88
Hg	0.24–3.95	0.02–9.88	0.24–4.04
As	3.12–10.70	0.02–5.85	2.82–7.27
Pb	0.29–1.24	0.46–1.17	0.47–1.24
Cr	0.35–0.80	0.38–0.74	0.38–0.77
Cu	0.92–2.54	0.75–2.08	0.67–2.69
Zn	0.18–0.48	0.19–0.48	0.14–0.42

## Data Availability

The data and R codes that support the findings of this study are available on request from the corresponding author.
